# Utility of dual time point 18 FDG PET-CT

**DOI:** 10.1186/1470-7330-14-S1-P24

**Published:** 2014-10-09

**Authors:** SE De Luca, JE Codas Thompson, E Casalini Vañek, H Campanelli, C Carrera, EP Eyheremendy

**Affiliations:** 1 Hospital Alemán, Buenos Aires, Argentina

## 

The Standardized Uptake Value (SUV) from FDG PET-CT is a simplified quantitative measure used for characterization of tissues suspected for malignancy. Its rationale is based in augmented glucose consumption of oncologic cells although, many other benign pathologies, could show elevated values leading to error.

The purpose of this presentation is to demonstrate that SUV value continues elevating in malignant pathology while in benign disease will decrease over time.

We prospectively acquired delayed images in patients with focal increase of metabolic activity without clear morphologic pathology, or failing to show increase metabolic activity in sites with morphological findings of probable oncologic origin. We have acquired delayed evaluation in 45 patients with suspicious lesions.

The most prominent findings were: 11 patients with focal hypermetabolic sites in gastrointestinal tract who demonstrate significant SUV decline on delayed images, avoiding unnecessary colonoscopies, while another 17 patients have significantly increased SUV on delayed images, correlated with polypoid lesions at colonoscopy. 7 patients with falling SUV on delayed images in adnexal hypermetabolic foci proved to be of functional origin at ultrasound follow-up, 5 patients with descending SUV at delayed images in hypermetabolic foci in the neck proved to be inflammatory on follow-up and 5 patients that SUV value continues elevating on delayed images, secondary or primary origin was confirmed.

Dual time point PET-CT has proven to be an important tool for proper characterization of questionable lesions that would lead to costly mistakes or unnecessary diagnostic studies.

